# IRESPred: Web Server for Prediction of Cellular and Viral Internal Ribosome Entry Site (IRES)

**DOI:** 10.1038/srep27436

**Published:** 2016-06-06

**Authors:** Pandurang Kolekar, Abhijeet Pataskar, Urmila Kulkarni-Kale, Jayanta Pal, Abhijeet Kulkarni

**Affiliations:** 1 Bioinformatics Centre, Savitribai Phule Pune University (Formerly University of Pune), Pune, Maharashtra, 411 007, India; 2 Department of Biotechnology, Savitribai Phule Pune University (Formerly University of Pune), Pune, Maharashtra, 411 007, India

## Abstract

Cellular mRNAs are predominantly translated in a cap-dependent manner. However, some viral and a subset of cellular mRNAs initiate their translation in a cap-independent manner. This requires presence of a structured RNA element, known as, Internal Ribosome Entry Site (IRES) in their 5′ untranslated regions (UTRs). Experimental demonstration of IRES in UTR remains a challenging task. Computational prediction of IRES merely based on sequence and structure conservation is also difficult, particularly for cellular IRES. A web server, IRESPred is developed for prediction of both viral and cellular IRES using Support Vector Machine (SVM). The predictive model was built using 35 features that are based on sequence and structural properties of UTRs and the probabilities of interactions between UTR and small subunit ribosomal proteins (SSRPs). The model was found to have 75.51% accuracy, 75.75% sensitivity, 75.25% specificity, 75.75% precision and Matthews Correlation Coefficient (MCC) of 0.51 in blind testing. IRESPred was found to perform better than the only available viral IRES prediction server, VIPS. The IRESPred server is freely available at http://bioinfo.net.in/IRESPred/.

Translation initiation in eukaryotes occurs principally in a 5′cap-dependent manner. For such initiation, the 40S ribosomal subunit along with eukaryotic initiation factors (eIFs) namely eIF1, eIF1A, eIF3, eIF5 and the ternary complex comprising eIF2 · GTP · Met-tRNA_i_ together forms the 43S pre-initiation complex. Then, the cap binding complex composed of eIF4A, eIF4E and eIF4G recruits the pre-initiation complex to the cap structure (m^7^G). The ribosomal subunit then scans the mRNA until it reaches start codon^1^. Alternatively, for some transcripts, translation can initiate internally in a cap-independent manner. An element present in 5′UTR of these messages, called IRES, is known to facilitate such internal initiation^2^.

Presence of IRES was first experimentally demonstrated in viruses of *Picornaviridae* family^3^,^4^. During viral infections and cytoplasmic stresses, global cellular translation is inhibited through a variety of mechanisms^1^. However, some viral and a subset of cellular transcripts encoding stress responsive and regulatory proteins, are translated through IRES mediated internal initiation mechanism. Therefore, IRES plays an important role in viral replication and development of integrated stress response. Moreover, IRES mediated initiation has also been reported during vital cellular processes such as mitotic cycle and apoptosis^5^,^6^. Similarly, dysfunction of IRES has also been linked to pathophysiological conditions^7^,^8^.

IRES element therefore, has been tested as a potential therapeutic target^9^. Although, such interventions are yet to be materialized extensively, demonstrating presence of these elements in 5′UTRs, still holds importance. Experimental methods available for detection of IRES presence require multiple cloning, transfection steps and appropriate controls^10^. The methods are multifaceted and thus, require skilled personnel. Further, the final outcome from such experimentation, depends on transfection efficiencies, cryptic promoter activity of query sequence(s) and presence of splice sites in them^11^. Therefore, attempts have been made to develop algorithms for computational prediction of IRES, which are expected to help experimentalists to narrow down the search space and thereby facilitate discovery of novel IRES.

*In silico* tools, such as IRSS and VIPS have been developed for viral IRES secondary structure prediction^12^,^13^ of which only VIPS is available online. These tools use structural conservation of IRES, which is observed in a few viral genera and perform prediction using structure comparison approach. However, prediction of cellular IRES remained intractable due to lack of conservation of sequence and/or structure. UTR features such as length, number of upstream AUGs, secondary structure complexity, folding energy etc. alone or in combination do not serve towards development of a reliable tool for prediction of cellular IRES^14^. Thus, there is a need to develop computational tool for prediction of IRES elements in 5′UTRs of cellular transcripts.

The state-of-the-art understanding of IRES and widely accepted theories specify that i) housekeeping gene messages, principally follow the cap dependent initiation pathway^15^ whereas, internal initiation process is confined to some viral, and cellular transcripts which encode regulatory and stress responsive proteins and ii) IRES element present in UTRs of these transcripts contests cap-dependent initiation for components of translation machinery including ribosomes^2^. However, recent studies indicate that the interplay between 40S ribosomal subunit proteins and IRES elements is crucial for IRES dependent initiation^2^,^16^,^17^,^18^,^19^,^20^. These studies are restricted to viral IRES (HCV and CrPV) and direct interactions between 40S ribosomal subunit proteins and cellular IRES elements have not yet been demonstrated experimentally. An indirect evidence of importance of ribosomal protein (through stable knock down of 40S ribosomal subunit protein) for cellular IRES mediated translation is available^21^. Although, individual 40S ribosomal subunit proteins are yet to be assigned with exact role in the internal initiation process, these studies indicated highlights the cross-talk between IRES element and the ribosomal proteins which cumulatively execute IRES mediated translation. Therefore, in the present study, an attempt has been made to utilize the interaction probabilities of 27 different small-subunit ribosomal proteins (SSRPs) with 5′UTR sequences by determining if these features possess information content to classify UTRs as IRES positive (IP) or negative (IN). In addition to these 27 features, we also used 8 general features of UTR to complement the classification. We have made a successful attempt to devise support vector machine (SVM) based classification model for the prediction of viral and cellular IRES using the features discussed above. The following sections describe the extraction of features, training and testing of SVM models, performance evaluation and its implementation in the form of web server, IRESPred. The comparison of IRESPred with VIPS, the only available server for prediction of viral IRES is also presented.

## Methods

### Compilation of data sets

The following true positive and negative data sets were compiled for training and testing of SVM based classification model.

#### Positive data set

IRESite (http://www.iresite.org) database archives experimentally characterised viral- and cellular-IRES elements. The database has archived 114 entries on its web portal. Whereas, supplementary information provided with the IRESite article^22^ enlists 183 entries. Therefore, the positive data set was compiled by considering entries from both the sources but by removing redundancy as well as challenged and synthetic IRES entries. Thus 114 and 75 entries were selected from IRESite web portal and supplementary data^22^, respectively. For every entry extracted from the IRESite, sequence of complete cellular 5′UTRs as annotated in the corresponding GenBank record was compiled. However, the sequence coordinate information as provided in respective IRESite entries was used to curate viral sequences. Similarly, during curation of entries from supplementary data, extraction of sequences was carried out either by referring the original articles or by searching the corresponding GenBank records. Of the total 189 IRES-positive sequences, there are 58 human, 58 other eukaryotic and 73 viral sequences.

### Negative data set

In the absence of availability of experimentally proven IRES-negative sequences, sequences of 5′ UTRs of housekeeping genes (*n* = 97), cellular CDS (*n* = 46) and viral CDS (*n* = 46), were randomly selected and compiled as a negative data set. The human housekeeping genes were selected from the recently published list^23^. The viral CDS were extracted from the records of viral genomes available in GenBank database^24^.

The details of positive and negative data sets are provided in Tables S1–S5 of Supplementary Data S1. The sequence data used in the present study will be made available upon request.

### Selection of features for classification

A total of 35 features were computed for each of the sequences in the positive and the negative data sets. These features include 8 general characteristics of UTRs namely length, number of upstream AUGs, number of hairpin-, external-, internal-, multi- and total-loops as observed in the predicted secondary structure of respective UTR and its folding energy. The remaining 27 features correspond to computationally predicted interaction probabilities of every UTR with each of the 27 SSRPs listed in Table S6 of Supplementary Data S1.

RNAFold 2.1.8 program in Vienna RNA package (http://rna.tbi.univie.ac.at/cgi-bin/RNAfold.cgi) was used to determine the number of loops mentioned above and folding energy of predicted secondary structure^25^. Whereas, interaction probabilities between UTR and SSRPs were predicted using the standalone version of RPISeq web server (http://pridb.gdcb.iastate.edu/RPISeq)^26^. Scripts were written in JAVA and PERL to automate extraction of these features using indicated resources, wherever necessary.

### Implementation of SVM models for classification

Support vector machine (SVM) is one of the most accurate machine learning algorithm and has been successfully used to address classification problems in Bioinformatics^27^,^28^. Therefore, in order to build and implement a model for classification of sequences as IP (IRES positive) or IN (IRES negative), SVM implemented in LibSVM 3.12 was used^29^. From the compiled data set consisting of 378 IRES positive and negative sequences, 5 training and test data sets were generated randomly such that every data set has equal proportion of positive and negative sequences. As a result, 50% of the total positive and negative sequences were used for training the model and the remaining 50% sequences were used for testing the same.

Thus, five models were built from 5 independent training data sets by using a set of scaled feature values derived from positive and negative sequences in the corresponding training data set. For each of the training data set, the SVM parameters such as SVM type (s), kernel type (t), degree (d), gamma (g) and cost (c) were optimised using swarm optimisation^29^. The model selection was carried out using the optimum set of parameters selected on the basis of 10 fold cross validation accuracy.

### Evaluation of SVM models

The predictive performance of models on test data sets was assessed using accuracy, sensitivity, specificity, precision and Matthews correlation coefficient (MCC) using following formulae (1–5).


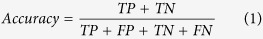



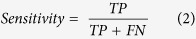



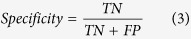



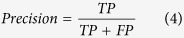



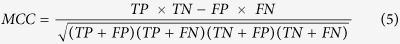


where,

TP: number of true positives

TN: number of true negatives

FP: number of false positives

FN: number of false negatives

The model showing optimum performance in terms of the measures described above was chosen for implementation as the web server for IRES prediction.

**Figure 1:**
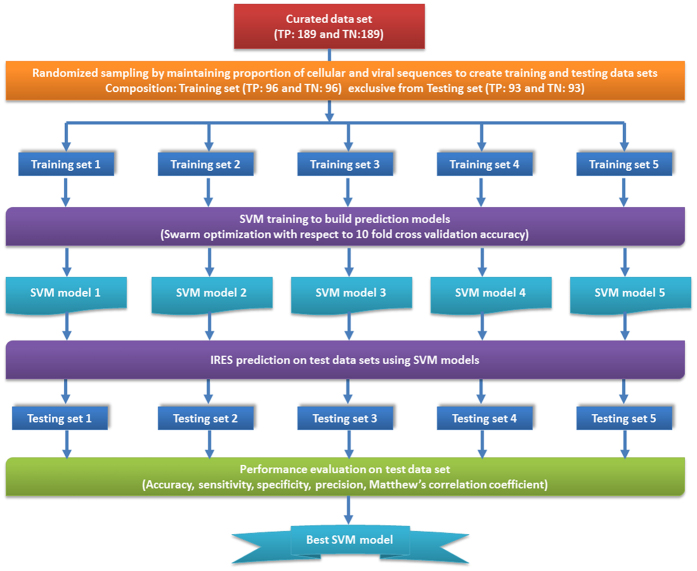
The strategy used for training and test data set generation, model building and evaluation.

The strategy for division of training and test data sets, model building and evaluation is depicted in Fig. 1.

### Development of the Web Server, IRESPred

The IRESPred web server for prediction of IP and IN sequences was developed using Apache, HTML, CSS, PHP and CGI. The coding and calculations required for predictions are generated using accessory JAVA and PERL programs. The server can be accessed online at http://bioinfo.net.in/IRESPred/.

### Comparison of IRESPred with VIPS server

The positive and negative sequences used in the present study were submitted to VIPS server for IRES prediction. The parameters, Group1-CrPV, Group2-HCV, Group3-EMCV, Group4-PV and Pseudoknot, as provided by VIPS server^13^ were turned on while performing predictions. The overall predictive performance of VIPS was evaluated using accuracy, sensitivity, specificity, precision and MCC values and compared with IRESPred server.

## Results

Internal ribosome entry sites (IRES), being potential therapeutic targets^9^, determining their presence in a given nucleotide stretch holds clinical importance. Experimental or computational methods capable of demonstrating the presence of IRES in sequences of interest will certainly help biologists working in the area of translation related diseases. The following sections describe the performance of IRESPred developed for cellular and viral IRES prediction.

### Building SVM models and performance evaluation

The comparative account of five independent SVM models built using swarm optimisation algorithm and evaluation of their performance using test data sets is given in Table 1. The similarities and differences observed in parameters and performance values of these models can be attributed towards compositional variations among the five training and test data sets. Further, the observation that models, which showed higher cross validation accuracy but inferior performance (e.g., model 4), and vice versa, illustrates an effect of bias, over fitting of data and noise during model building. The best performing SVM model (model 1) with accuracy, sensitivity, specificity, precision and MCC of 75.51%, 75.75%, 75.25%, 75.75% and 0.51, respectively has been implemented in IRESPred web server.

### IRESPred Web server

**Figure 2:**
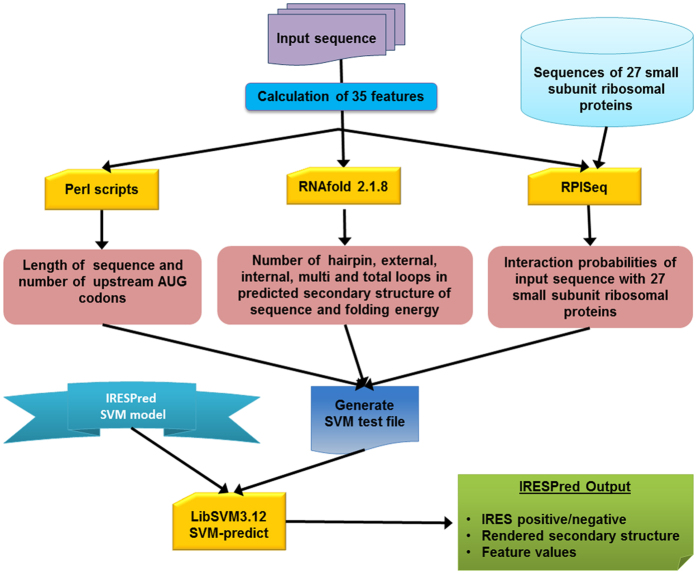
The process flow of IRESPred web server.

The IRESPred web server implemented with SVM model 1, as described in previous section, is made available at http://bioinfo.net.in/IRESPred/. The server accepts up to 10 nucleotide sequence(s) having lengths between 15 and 7500 bases in fasta format. The restriction on length of input sequences is due to the limits set by RNAfold and RPISeq programs used in backend processing of server. The submission of sequence(s) invokes the accessary programs to compute the values of 35 features for every input query sequence. The scaled feature values of sequence(s) are compiled in an appropriate format for IRES prediction using SVM-predict program and SVM model. The process flow of IRESPred web server is depicted in Fig. 2. The server provides the IRES prediction either as IP or IN, rendered image of secondary structure and values of 35 features. IRESPred is a general purpose tool that supports both, cellular and viral IRES prediction.

### Comparison of IRESPred with VIPS server

The VIPS server was originally developed for prediction of four viral IRES groups namely *Cricket Paralysis virus* (CrPV), *Hepatitis C virus* (HCV), *Encephalomyocarditis virus* (EMCV) and *Polio virus* (PV) groups^13^. The developers of VIPS have also tested its performance on human 5′UTRs. Therefore, sequences from positive and negative data sets (of both viral and cellular origin) used in the present study were subjected to IRES predictions using both the servers, IRESPred and VIPS, to compare their performances. The performance comparison is summarised in Table 2. The results indicated accuracy of 70.89% and 51.87% for IRESPred and VIPS respectively, with IRESPred showing overall better performance than VIPS. Thus, IRESPred provides a better alternative not only for viral but also for cellular IRES prediction.

### Performance of IRESPred for known Viral & cellular IRES: A comparison

The performance of the IRESPred was tested on experimentally validated viral (*n* = 73) and cellular (human *n* = 58 and other *n* =  58) IRES sequences. The server could correctly predict 67 viral IRES and achieved 91.8% accuracy for prediction of viral IRES. Similarly, out of 116 experimentally verified cellular IRES sequences, the server could correctly predict 85 sequences indicating an accuracy of 73.2%. Evaluation of the server for human IRES sequences revealed that the server could correctly predict 44 sequences and performed with an accuracy of 75.86%. The authors of VIPS have performed similar study for known human IRES sequences and reported an accuracy of 21.98%^13^. Thus, the IRESPred server performs significantly better than VIPS server for prediction of cellular IRES and is expected to be a suitable alternative for prediction of both, viral and cellular IRES.

Recently 5′UTR of human DDB2 mRNA, which encodes the tumor-inhibiting factor involved in DNA repair and DNA damage induced apoptosis, has been shown to harbour an IRES^30^. Being published very recently, DDB2 entry was not archived in IRESite and thus was not part of training and testing data sets in the present study. When tested independently, IRESPred successfully predicted DDB2 5′UTR as IP, which substantiates the predictive ability of IRESPred server for cellular IRES.

## Discussion

Internal ribosome entry sites (IRES) play a vital role in cellular physiology and stress response through translation of specific viral or stress responsive cellular mRNAs. Therefore, IRES have been tested as therapeutic targets. Discovery of these elements in novel viral and cellular mRNAs thus hold a great importance. Conventional experimental protocols available for demonstration of these elements require *in vitro* amplification of specific elements, their cloning in mono-cistronic/bi-cistronic constructs and transfection of such recombinant vectors to mammalian cells followed by determination of expression levels of reporter genes. Alternatively, the query sequence containing transcripts are circularized and their capability to get translated is monitored. The determination of exact location of IRES (core element) requires deletion studies. Thus, these experimental methods are tricky, laborious and require skilled personnel. The outcome of experiment still depends on many more factors^11^.

Therefore, computational prediction of IRES will help molecular biologists to narrow down on potential sequences to be validated experimentally. However, computational methods are based on conservation of certain properties and/or parameters to devise predictive models, which have been tried in case of viral IRES prediction. The publicly available VIPS server uses structural conservation observed in four viral IRES groups to perform predictions. Since, cellular IRES lacks conservation in structural and sequence related parameters; no computational method has been developed so far for prediction of the cellular IRES. Therefore, prediction of cellular IRES still remains the challenging task for computational biologists. The availability of true positive and negative data sets plays a deterministic role in development of predictive model. The published IRES sequences archived in IRESite database were used as positive data set in the present study. In the absence of experimentally validated true negative data, the sequences of the housekeeping genes, which principally follow cap-dependent initiation pathway^15^, were considered to be the appropriate set to serve as true negative sequences. Similarly, IRES being a characteristic feature of UTR regions, the downstream coding regions are expected to be devoid of this property. Therefore, the compiled negative data set include 5′ UTRs of housekeeping genes, their coding sequences and viral coding sequences.

The extraction of relevant features from the data set entries then determines success of predictive model. The UTR features such as length, number of upstream AUGs, secondary structure complexity, folding energy etc., are known and observed to differ in IP and IN sequences. However, these features alone or in combination are reported to be insufficient for development of a reliable IRES prediction tool^14^. Therefore, additional complementary features having information content were required towards development of a predictive model.

For internal initiation process, IRES elements compete cap-dependent initiation for components of translation machinery including ribosomes is an accepted hypothesis. However, the interplay between conformation of IRES and ribosome is thought to be more relevant^2^,^16^,^17^,^18^,^19^,^20^. Therefore, interaction between IRES and ribosomal proteins is an essential step for successful internal initiation. In view of these facts, interaction probabilities between UTRs and all SSRPs were tested as additional features. We observed that interaction probabilities between UTRs and 27 SSRPs were informative and are used in predictive model development for IRES prediction. The RPISeq being the partner specific RNA-protein interaction prediction tool, has been used to calculate the UTR-SSRPs interaction probabilities^26^. Using combination of these features, IRESPred was found to perform better than VIPS and hence it provides a suitable alternative for viral IRES prediction. Further, IRESPred has been specifically developed for prediction of both, viral and cellular IRES. The ribosomal proteins have high level of sequence similarity across species therefore the predictive model implemented in IRESPred uses human ribosomal proteins only. However, usage of host specific (for viral IRES) and species specific (for cellular IRES) SSRP sequences will certainly improve the predictive performance of IRESPred and the development of such models is under process and will be made available in the forthcoming versions of the IRESPred server.

Thus, the principle parameter used to develop the IRES prediction method and the IRESPred server, is the interaction between 40S ribosomal proteins and IRES sequences. Experimentally demonstrated using viral IRES (HCV and CrPV), these interactions have been extrapolated for prediction of cellular IRES in the present study. As evident from the validation studies, the method in its current form is found to work satisfactorily for prediction of both, viral and cellular IRES. However, use of the method for prediction of cellular IRES comes with a caveat that the ribosomal protein-IRES interactions used as a predictor in the present study, have not been demonstrated experimentally for cellular IRES elements. Further, viral IRES (HCV and CrPV in particular) are atypical IRES and have minimal or no requirement for initiation factors and therefore are not comparable to cellular IRES. Despite the caveat, it is envisaged that the IRESPred server will accelerate the pace of research for characterisation of both, viral and cellular IRES.

## Conclusions

An algorithm and a web server titled IRESPred, is designed, developed and validated for prediction of viral and cellular IRES. The IRES driven internal initiation mechanism is intrinsically complex yet very important for normal cellular physiology. The IRESPred will help molecular biologists to choose potential target genes containing putative IRES for further experimentation. Thus, IRESPred server is expected to fill the gap for the researchers engaged in translation regulation of gene expression.

## Additional Information

**How to cite this article**: Kolekar, P. *et al*. IRESPred: Web Server for Prediction of Cellular and Viral Internal Ribosome Entry Site (IRES). *Sci. Rep*. **6**, 27436; doi: 10.1038/srep27436 (2016).

## Supplementary Material

Supplementary Information

## Figures and Tables

**Table 1:** The optimum parameters employed in model building using training sets and performance evaluation using testing sets.

Model	Optimum parameters used in model building^*^						Performance measures used in model evaluation^§^				
	s	t	d	g	c	CV (%)	Acc (%)	Sn (%)	Sp (%)	Pr (%)	MCC
1	2	0	1	3.1192	0.0347	63.54	75.51	75.75	75.25	75.75	0.51
2	1	1	1	1.8022	0.0347	68.75	63.44	63.44	63.44	63.44	0.26
3	0	1	2	1.0050	1.0397	67.19	62.36	62.36	62.36	62.36	0.24
4	2	0	1	3.1192	0.0347	69.79	65.05	61.30	68.82	66.28	0.30
5	1	1	1	2.2181	0.0347	67.71	61.83	60.22	63.44	62.22	0.23

^*^s: SVM type, t: kernel type, d: degree, g: gamma, c: cost and CV: 10-fold cross validation accuracy. Parameters as specified by svm-train program in LibSVM3.12 package.

^§^Acc: accuracy, Sn: sensitivity, Sp: specificity, Pr: precision and MCC: Matthews correlation coefficient.

**Table 2:** The performance comparison of IRESPred and VIPS servers using positive and negative data sets compiled in the present study.

Server	Accuracy (%)	Sensitivity (%)	Specificity (%)	Precision (%)	MCC
IRESPred	70.89	69.84	71.95	71.35	0.41
VIPS	51.87	23.28	81.08	55.69	0.053
