# Supplementary material for: IRESPred: Web Server for Prediction of Cellular and Viral Internal Ribosome Entry Site (IRES)
Source: Sci Rep. 2016 Jun 6;6:27436. doi: 10.1038/srep27436 (PMC4893748; doi:10.1038/srep27436)
Supplement: Supplementary Information [file srep27436-s1.pdf]

IRESPred: Web Server for Prediction of Cellular and Viral Internal Ribosome Entry Site (IRES)

Pandurang Kolekar, Abhijeet Pataskar, Urmila Kulkarni-Kale, Jayanta Pal and Abhijeet Kulkarni

Supplementary data legends:

Supplementary Data S1

The details of positive (Tables S1-S2) & negative data sets (Tables S3-S5) and list of 27 small subunit ribosomal proteins (Table S6) used in the present study.

Supplementary Data S1

Data sets

Positive data set consists of a total of 189 viral and cellular 5’UTR sequences given in Table S1 and S2.

Negative data set consists of a total of 189 viral and cellular gene coding sequences and 5’UTRs of cellular housekeeping genes. Details of negative data sets are provided in Table S3, S4 and S5.

List of 27 small subunit ribosomal proteins (SSRP) is given in Table S6.

Table S1: Positive data set: Viral entries (Total: 73)

Sr. No.	Virus	IRESite ID[1]	GenBank accession No.[2]
1	Drosophila C virus strain EB	621	AF014388.1
2	Blackcurrant reversion virus	N/A	AF020051.3
3	Rhopalosiphum padi virus	437	AF022937.1 (region: 1-579)
4	Rhopalosiphum padi virus	437	AF022937.1 (region: 6875-7106)
5	Rous sarcoma virus	N/A	AF033808.1
6	Human immunodeficiency virus 2	N/A	AF082339.1
7	Triatoma virus	626	AF178440.1 (region1-694)
8	Triatoma virus	626	AF178440.1 (region: :5929-6149)
9	Taura syndrome virus	77	AF277675.1
10	Porcine enterovirus 8	N/A	AF406813.1
11	Human echovirus 6	N/A	AF465517.1
12	Foot-and-mouth disease virus	321	AJ133357.1
13	Hepatitis GB virus B	54	AJ277947.1
14	Turnip mosaic virus	N/A	AY227024.1
15	Human echovirus 25	N/A	AY302549.1
16	Youcai mosaic virus	39	AY318866.1 (region: 4649-4876)
17	Youcai mosaic virus	39	AY318866.1 (region: 5456-5601)
18	Human coxsackievirus B3	225	AY752946.1
19	Murine hepatitis virus	N/A	AY910861.1
20	Potato leaf roll virus	N/A	D13954.1
21	Human enterovirus 71	637	DQ060149.1
22	Homalodisca coagulata virus-1	622	DQ288865.1
23	Reticuloendotheliosis virus	435	DQ387450.1
24	Poliovirus	598	K01392.1
25	Infectious bronchitis virus	N/A	KC506155.1
26	Hibiscus chlorotic ringspot virus	N/A	KC876666.1
27	Giardia lamblia virus	N/A	L13218.1
28	Equine rhinovirus 1	286	L43052.1
29	Hepatitis A virus	42	M14707.1
30	Tobacco etch virus	N/A	M15239.1
31	Theiler's murine encephalomyelitis virus	597	M16020.1

32	Coxsackievirus B1	N/A	M16560.1
33	Simian (macaque) immunodeficiency virus	N/A	M19499.1
34	Hepatitis C virus subtype 1a	222	M67463.1
35	Bovine viral diarrhea virus 1	37	NC_001461.1
36	Encephalomyocarditis virus	140	NC_001479.1
37	Feline immunodeficiency virus	N/A	NC_001482.1
38	Human immunodeficiency virus 1	73	NC_001802.1
39	Bovine enterovirus	N/A	NC_001859.1
40	Perina nuda virus	N/A	NC_003113.1
41	Plautia stali intestine virus	59	NC_003779.1
42	Cricket paralysis virus	40	NC_003924.1 (region: 1-708)
43	Cricket paralysis virus	40	NC_003924.1 (region: 6025-6216)
44	Avian encephalomyelitis virus	416	NC_003990.1
45	Ectropis obliqua picorna-like virus	627	NC_005092.1
46	Hepatitis GB virus A	38	U22303.1
47	Hepatitis GB virus C	41	U36380.1
48	Feline leukemia virus	577	AB818696.1
49	Human poliovirus 1	242	V01149.1
50	Tobacco mosaic virus	615	V01408.1
51	Human rhinovirus 2	139	X02316.1
52	Friend murine leukemia virus	N/A	X02794.1 (region: 1-621)
53	Potatovirus	N/A	X12456.1
54	Echovirus 11	N/A	X80059
55	Equine Rhinovirus type 2	26	X96871.1
56	Turnip vein-clearing virus	603	Z29370.1 (region: 26-166)
57	Epstein-Barr virus	N/A	S45894 (region: 465-608)
58	Drosophila melanogaster gypsy transposable element	69	M12927.1 (region: 1-330)
59	Drosophila melanogaster gypsy transposable element	69	M12927.1 (region: 530-790)
60	Human herpesvirus 8	N/A	U75698.1 (region: 122973-123206)
61	Human parechovirus 1	N/A	EF051629.2 (region: 298-538)
62	Human herpesvirus 1	N/A	FJ655111.1 (region: 535-573)
63	Porcine teschovirus 1	N/A	AF231769.1 (region: 1-432)
64	Drosophila melanogaster, Idefix retroelement (gag, pol and env)	N/A	AJ009736.1
65	Gallid herpesvirus 2	N/A	AF243438.1 (region: 131117-131361)
66	Murid herpesvirus 4	N/A	NC_001826.2 (region: 25330-25715
67	Moloney murine leukemia virus	N/A	J02255.1 (region: 495-621)
68	Simian sapelovirus 1	N/A	AY064708.1 (region: 253-746)
69	Swine vesicular disease virus	N/A	AY429470.1 (region: 69-635)
70	Mouse DNA for virus-like (VL30) retrotransposon BVL-1	N/A	X51336 (region: 462-1144)
71	White spot syndrome virus	N/A	AF227911.1 (region: 303-482)
72	Turnip vein-clearing virus	603	Z29370.1 (region: 655-795)
73	Hog cholera virus (Classical swine fever virus)	148	Z46258.1

Note: The entries having no IRESite ID were taken from supplementary file provided with Mokrejs et al, (2010)[1].

Table S2: Positive data set: Cellular 5'UTR entries (Total: 116)

Sr. No.	Organism and gene name	IRESite ID[1]	GenBank accession No.[2]
1	Drosophila melanogaster, antennapedia (Antp)	71	NM_206445.1
2	Mus musculus, apoptotic protease activating factor 1 (Apaf-1)	342	AF064071.1
3	Homo sapiens, apoptotic protease activating factor 1 (Apaf-1)	110	AK307509.1
4	Homo sapiens, (APC)	N/A	M74088.1
5	Homo sapiens, amyloid beta (A4) precursor protein (APP)	N/A	NM_000484.3
6	Homo sapiens, mercurial-insensitive water channel (AQP4)	491	U34845.1
7	Homo sapiens, angiotensin II receptor (AT1R)	N/A	NM_031850.3
8	Homo sapiens, BCL2-associated athanogene (BAG1)	N/A	NM_004323.5
9	Homo sapiens, bcl-2-alpha protein (bcl-2)	103	M13994.1
10	Homo sapiens, BiP protein (BiP)	570	X87949.1
11	Homo sapiens, baculoviral IAP repeat containing 2 (BIRC2)	N/A	NM_001166.4
12	Homo sapiens, v-myb avian myeloblastosis viral oncogene homolog (C-MYB)	471	NM_005375.2
13	Homo sapiens, c-myc oncogene (C-MYC)	35	V00568.1
14	Rattus norvegicus, cationinc amino acid transporter 1 (Cat1)	438	AF245000.1
15	Homo sapiens cyclin D1 (CCND1)	258	NM_053056.2
16	Homo sapiens, cold inducible RNA binding protein (CIRBP)	N/A	NM_001300829.1
17	Homo sapiens, connexin 26 (cx26)	N/A	U43932.1
18	Homo sapiens, (CYR61)	N/A	Y11307.1
19	Homo sapiens, eukaryotic translation initiation factor 4 gamma (DAP5)	117	NM_001418.3
20	Homo sapiens, (P73)	N/A	Y11416
21	Homo sapiens, eukaryotic initiation factor 4 gamma (eIF4G)	548	D12686.1
22	Homo sapiens, eukaryotic translation initiation factor 4 gamma (eIF4GI)	N/A	NM_182917.4
23	Homo sapiens, (eIF4GII)	573	AF012072.2
24	Homo sapiens, cDNA FLJ43058 fis (ELG1)	492	AK125048.1
25	Homo sapiens, clone UGL16c06 (FGF1)	519	DQ655917.2
26	Homo sapiens, (FGF2)	N/A	X04431.1
27	Homo sapiens, fragile X mental retardation 1 (FMR1)	N/A	NM_002024.5
28	Homo sapiens, protein disulfide isomerase family A (PDIA3, GRP58)	N/A	NM_005313.4
29	Mus musculus, (GTX)	204	L08074.1
30	Drosophila melanogaster, mRNA for hairless serine rich protein (hairless)	66	X67239.1
31	Mus musculus, hypoxia inducible factor 1 (Hif1a)	N/A	NM_010431.2
32	Zea mays, heat shock protein HSP101 (HSP101)	46	AF133840.1
33	Drosophila melanogaster, Heat-shock-protein-70Aa (Hsp70Aa)	N/A	NM_169441.2
34	Drosophila melanogaster, heat shock protein 83 (Hsp83, Hsp90)	N/A	NM_079175.3
35	Homo sapiens, insulin-like growth factor IGFII gene leader exon (IGF2)	337	X53038.1
36	Homo sapiens, interferon regulatory factor 2 (IRF2)	N/A	NM_002199.3
37	Homo sapiens, v-myc avian myelocytomatosis viral oncogene lung carcinoma derived homolog (MYCL, L-MYC)	N/A	NM_001033082.2
38	Homo sapiens, laminin (LamB1)	583	NM_002291.2
39	Homo sapiens, lymphoid enhancer-binding factor 1 (LEF1)	N/A	NM_016269.4

40	Homo sapiens, MAX network transcriptional repressor (MNT)	51	NM_020310.2
41	Homo sapiens,leucine zipper protein 6 (LUZP6, MPD6)	N/A	NM_001128619.2
42	Homo sapiens, runt-related transcription factor 1 (RUNX1T1)	N/A	NM_004349.3
43	Homo sapiens, myelin transcription factor 2 (MYT2)	49	AF006822.1
44	Homo sapiens, v-myc avian myelocytomatosis viral oncogene neuroblastoma derived homolog (MYCN, n-MYC)	N/A	NM_005378.5
45	Homo sapiens, nucleosome assembly protein (NAP1L1)	N/A	D28430.1
46	Mus musculus, N-deacetylase/N-sulfotransferase (heparan glucosaminyl) 1 (Ndst1)	287	NM_008306.4
47	Mus musculus, N-deacetylase/N-sulfotransferase (heparan glucosaminyl) 2 (Ndst2)	288	NM_010811.2
48	Mus musculus, N-deacetylase/N-sulfotransferase (heparan glucosaminyl) 3 (Ndst3)	N/A	NM_031186.3
49	Mus musculus, N-deacetylase/N-sulfotransferase (heparin glucosaminyl) 4 (Ndst4)	N/A	NM_022565.2
50	Homo sapiens, nucleophosmin, nucleolar phosphoprotein B23 (NPM1)	N/A	NM_002520.6
51	Homo sapiens, transcription factor NRF (NRF)	243	AJ011812.2
52	Nicotiana tabacum, heat shock factor (NtHSF1)	612	AB014483.1
53	Homo sapiens, POU class 5 homeobox 1 (POU5F1, OCT4B)	N/A	NM_001285986.1
54	Rattus norvegicus, ornithine decarboxylase (ODC1)	107	M16982.1
55	Homo sapiens, opioid receptor (OPRM1)	N/A	NM_001285524.1
56	Homo sapiens, (p53)	599	AF307851.1
57	Homo sapiens, c-sis/platelet-derived growth factor gene (PDGF2)	102	M19719.1
58	Homo sapiens, protein kinase PITSLRE alpha 2-2 (P58PITSLRE)	436	U04816.1
59	Mus musculus, RNA-binding motif protein 3 (Rbm3)	8	AY052560.1
60	Drosophila melanogaster, reaper (rpr)	N/A	NM_079414.3
61	Homo sapiens, runt-related transcription factor 1 (RUNX1)	N/A	NM_001001890.2
62	Canis familiaris, scamper (scamper)	111	AF263546.2
63	Homo sapiens, septin 9 (SEPT9)	N/A	NM_001113492.1
64	Homo sapiens, SMAD5 splice variant B (SMAD5)	N/A	AF071107.1
65	Homo sapiens, surfactant protein A2 (SPA2)	N/A	BC157890.1
66	Homo sapiens, microtubule-associated protein tau (MAPT, TAU)	N/A	NM_016835.4
67	Saccharomyces cerevisiae, TATA-box factor (TFIID)	629	M26403.1
68	Homo sapiens, thrombomodulin (THBD)	N/A	NM_000361.2 (region: 1-160
69	Saccharomyces cerevisiae, CAP-binding protein complex subunit p150 (TIF4631)	572	L16923.1
70	Homo sapiens, neurotrophin receptor tyrosine kinase type 2 (NTRK2, TRKB) gene	N/A	AF410902.1
71	Drosophila melanogaster, Ultrabithorax (Ubx)	65	BT010241.1
72	Homo sapiens, cold shock domain containing E1 (CSDE1, UNR)	N/A	NM_001007553.2
73	Mus musculus, vascular endothelial growth factor A (Vegfa)	571	NM_001025257.3
74	Homo sapiens, (XIAP)	N/A	BX119811.1
75	Saccharomyces cerevisiae, SNQ3 gene confering mutagen hyper-resistance (SNQ3)	628	X63268.1
76	Saccharomyces cerevisiae, chromosome XI reading frame ORF (YKL109w)	116	Z28109.1
77	Homo sapiens, KIAA0086 (hSNM1)	58	D42045.1
78	Homo sapiens, heat shock 70kDa protein 1A (Hsp70, HSPA1A)	118	NM_005345.5
79	Mus musculus, potassium voltage-gated channel, shaker-related subfamily, member 4 (Kcna4)	124	NM_021275.3

80	Saccharomyces cerevisiae strain CBS5112 Ure2p (URE2)	115	AF525191.1
81	Rattus norvegicus calcium/calmodulin-dependent protein kinase II alpha (Camk2a)	N/A	NM_012920 (1-41)
82	Drosophila melanogaster Adh-related (Adhr), transcript variant B	N/A	NM_001032101 (region: 844-1146
83	Rattus norvegicus activity-regulated cytoskeleton-associated protein (Arc)	N/A	NM_019361 (region: 1-216
84	Mus musculus betaPix-b mRNA	N/A	AF247654.1 (region: 1-303
85	Mus musculus Bcl-xL	N/A	L35049.1 (region: 1-242
86	Saccharomyces cerevisiae Bem1p-interacting protein (BOI1)	N/A	L31406.1 (region: 1-487
87	Mus musculus Cx32 gene for connexion (Cx32)	N/A	AJ271753.1 (region: 7081-7552
88	Rattus norvegicus gap junction protein, alpha 1 (Gja1)	N/A	NM_012567.2 (region: 1-196
89	Mus musculus E2F6 (E2f6) gene	N/A	AF393244S1 (region: 1571-2199
90	Aplysia californica egg-laying hormone (ELH)	N/A	NM_001204741.1 (region: 1-279
91	Saccharomyces cerevisiae (FLO8)	N/A	U51431.1 (region: 1-183
92	Saccharomyces cerevisiae (GIC1)	N/A	BK006934.2 (region: 222479-222672
93	Rattus norvegicus, glutamate receptor (Gria2)	N/A	NM_001083811.1 (region: 1-430
94	Saccharomyces cerevisiae, G protein coupled receptor (GPR1)	N/A	BK006938.2 (region: 392058-392457
95	Drosophila melanogaster grim (grim)	N/A	NM_079413.3 (region: 1-318
96	Drosophila melanogaster (hid)	N/A	NM_079412.4 (region: 1-519
97	Rattus norvegicus, insulin-like growth factor I (IGFI-R) receptor	N/A	M37807.1 (region: 416-1355
98	Drosophila melanogaster Insulin-like receptor (InR)	N/A	NM_001144622.2 (region: 1-419
99	Homo sapiens insulin receptor (INSR)	N/A	M76592.1 (region: 39-575
100	Gallus gallus jun proto-oncogene (JUN)	N/A	NM_001031289.1 (region: 1-313
101	Homo sapiens Sjogren syndrome antigen B (autoantigen La1)	N/A	NM_001294145.1 (region: 1-498
102	Rattus norvegicus microtubule-associated protein 2 (Map2)	N/A	NM_013066.1 (region: 1-102
103	Homo sapiens methionine synthase (MS)	N/A	U73338.1 (region: 1-394
104	Saccharomyces cerevisiae (MSN1)	N/A	BK006948.2 (region: 99467-99808
105	Saccharomyces cerevisiae (NCE102)	N/A	CP006243.1 (region: 806383-806840
106	Mus musculus NK6 homeobox 1 (Nkx6-1)	N/A	NM_144955.2 (region: 1-477
107	Rattus norvegicus protein kinase C, delta (PKCD)	N/A	BC076505.1 (region: 1-188
108	Arabidopsis thaliana 40S ribosomal protein S18 (RPS18C)	N/A	NM_117048.3 (region: 20-103
109	Rattus norvegicus neurogranin/RC3 protein (RC3)	N/A	U22062.1 (region: 4217-4475
110	Oryctolagus cuniculus (RGR)	N/A	Hernandez-Munoz et al., 2003*
111	Mus musculus ring finger protein 2 (Rnf2, Ring1b)	N/A	XM_006529269.1 (region: 53-205
112	Homo sapiens soluble guanylyl cyclase subunit beta 2 (GUCY1B2)	N/A	AF038499.2 (region: 1-280
113	Rattus norvegicus gene for V1b vasopressin receptor (V1br)	N/A	AB042197.1:join(4094-4125, 4288-4894)
114	Saccharomyces cerevisiae, (YMR181c)	N/A	CP005424.1 (region: 595521-595819
115	Rattus norvegicus dendrin (Ddn)	N/A	NM_030993.1 (region: 1-148
116	Mus musculus utrophin (Utrn)	223	NM_011682.4

Note: The entries having no IRESITE ID were taken from supplementary file provided with Mokrejs et al, (2010).

Table S3: Negative data set: Viral coding sequences (Total: 46)

Sr. No.	Virus	Gene	RefSeq Accession No.[2]	Genome Position
1	Achimota virus 1	F	NC_025403.1	4907-6508
2	Avian leukemia virus	env	NC_015116.1	593-2698
3	Avian paramyxovirus 3 strain turkey/Wisconsin/68	NZ89_gp2	NC_025373.1	1752-2924
4	Bovine adenovirus D	BAV4gp11	NC_002685.2	12031-13383
5	Cygnus olor circovirus isolate H51	NF97_gp2	NC_025247.1	968-1723
6	Cyprinid herpesvirus 3	CyHV3_ORF97	NC_009127.1	177739-180861
7	Human T-lymphotropic virus 1	gp46_SU	NC_001436.1	4829-6295
8	Ovine adenovirus D	OaV7gp08	NC_004037.2	9159-10166
9	Reticuloendotheliosis virus	gag	NC_006934.1	941-2443
10	Sulfolobus virus STSV2	STSV2_26	NC_020077.1	24628-26034
11	Turkey adenovirus A	52K	NC_001958.1	8569-9471
12	Vibrio phage VSK	VSKP12	NC_003327.2	5023-5460
13	Ranid herpesvirus 1	RaHV1_gp034	NC_008211	70899-73427
14	Cowpea chlorotic mottle virus	CCMVs1gp1	NC_003543	71-2947
15	Torque teno virus 1	orf2	NC_002076	353-3077
16	Acute bee paralysis virus	ABPVgp1	NC_002548	605-6325
17	Pineapple mealybug wilt-associated virus 1	RdRp	NC_010178	6568-8145
18	Blueberry mosaic associated virus	IR03_gp1	NC_024476	318-1865
19	Botrytis cinerea debilitation-related virus	RDRP	NC_011372	477-2066
20	Rabbit hemorrhagic disease virus	RHDVgp2	NC_001543	7025-7378
21	Spodoptera frugiperda ascovirus 1a	ORF027	NC_008361	31405-33420
22	Avian bornavirus	P	NC_024296	1254-1862
23	Heterocapsa circularisquama RNA virus	HcRNAV34ORF-1	NC_007518	19-3018
24	Equine arteritis virus	1ab	NC_002532	225-5408
25	Borna disease virus	G	NC_001607	2236-3747
26	Torque teno canis virus	TTcaV_gp2	NC_014071	662-967
27	Acidianus filamentous virus 3	AFV3_gp55	NC_010155	32633-33514
28	Marburg marburgvirus	NP	NC_024781	104-2191
29	Olive latent virus 1	OLV1gp1	NC_001721	61-2232
30	Pseudomonas phage	phi-6S_1	NC_003714	305-754
31	Adoxophyes orana granulovirus	granulin	NC_005038	1-747
32	Helminthosporium victoriae 145S virus	HVV145Ss4gp1	NC_005981	413-2554
33	Beak and feather disease virus	V2	NC_001944	550-1026
34	Bombyx mori densovirus 3	K707_sVD1gp1	NC_020928	311-691
35	Rhesus monkey papillomavirus	E2	NC_001678	2757-3857
36	Anatid herpesvirus 1	UL42	NC_013036	27152-28243
37	Anguillid herpesvirus 1	AngHV1_ORF30	NC_013668	43256-46450
38	Aedes taeniorhynchus iridescent virus	MIV014L	NC_008187	21706-23106
39	Acidianus two-tailed virus	ATV_gp50	NC_007409	28800-34622

40	Cauliflower mosaic virus	CaMVgp5	NC_001497	2201-3670
41	Nudaurelia capensis beta virus	Ncbvgp2	NC_001990	4039-5877
42	Acidianus bottle-shaped virus	ORF315	NC_009452	8759-9706
43	Chicken astrovirus	ANVgp1	NC_003790	14-4551
44	Ground squirrel hepatitis virus	GSHVgp1	NC_001484	1-654
45	Lettuce necrotic yellows virus	G	NC_007642	4412-6247
46	Bovine adenovirus 6	G355_gp06	NC_020074	4257-7478

Table S4: Negative data set: Cellular coding sequences (Homo sapiens) retrieved from UCSC genome browser (Assembly hg19) (Total: 46)

Sr. No.	Gene name	UCSC ID[3]	Genome position
1	ADSL	uc003ays.4	chr22:40742563-40762526
2	AGGF1	uc003ket.3	chr5:76326592-76359077
3	AGPAT1	uc003oab.1	chr6:32133943-32135733
4	AGPS	uc010zfb.1	chr2:178285007-178402923
5	AHCY	uc002xai.3	chr20:32868840-32891076
6	AK2	uc001bwq.2	chr1:33476430-33490117
7	ALAD	uc011lxe.2	chr9:116150580-116155839
8	AMBRA1	uc010rgt.2	chr11:46419490-46564264
9	ANAPC10	uc003ijx.3	chr4:146025565-146048725
10	ANO6	uc001rom.2	chr12:45566973-45568148
11	ANXA6	uc011dcp.2	chr5:150481009-150519726
12	AP2M1	uc003fmw.3	chr3:183894782-183901404
13	AKR7A2	uc001bbw.3	chr1:19630719-19638618
14	ZZZ3	uc001dhr.3	chr1:78031325-78105156
15	ENSA	uc001eve.3	chr1:150595320-150601946
16	ARNT	uc001evr.2	chr1:150784497-150849043
17	APOA1BP	uc001fpk.3	chr1:156562175-156563876
18	ARV1	uc001huh.3	chr1:231114852-231133009
19	MCU	uc001jtd.3	chr10:74452822-74645580
20	PLEKHA8P1	uc001rom.2	chr12:45566973-45568148
21	ALKBH1	uc001xuc.1	chr14:78140155-78174347
22	ECI1	uc002cps.3	chr16:2289980-2301567
23	SPAG7	uc002gae.3	chr17:4862829-4871099
24	ALYREF	uc002kbu.2	chr17:79846007-79849456
25	AES	uc002lwy.1	chr19:3053817-3062198
26	GPI	uc002nvi.2	chr19:34884921-34890941
27	PSMD8	uc002oii.4	chr19:38865242-38874030
28	LPIN1	uc002rbs.4	chr2:11881546-11924052
29	AFTPH	uc002scz.3	chr2:64778609-64819169
30	AGFG1	uc002vpd.2	chr2:228337138-228419211
31	C21orf33	uc002zed.4	chr21:45553580-45564831
32	SNRPD3	uc003aam.1	chr22:24953643-24967945
33	AP1B1	uc003afh.3	chr22:29726181-29727805

34	COX7C	uc003kir.3	chr5:85913873-85915286
35	AMD1	uc003pul.1	chr6:111213381-111214819
36	MEPCE	uc003uuv.3	chr7:100029049-100031177
37	FSCN3	uc003vmc.1	chr7:127231997-127236056
38	ATP6AP1	uc004flh.1	chrX:153657353-153664237
39	CAPZB	uc009vpk.3	chr1:19666061-19775408
40	APH1A	uc010pbz.2	chr1:150238922-150240125
41	AKIP1	uc010rbs.2	chr11:8932997-8939013
42	AHSA1	uc010tvk.1	chr14:77924533-77934955
43	RING1	uc011dqw.1	chr6:33176600-33176962
44	ARAF	uc011mlo.3	chrX:47424442-47428431
45	AKR1A1	uc021omx.1	chr1:46027467-46035628
46	ASXL1	uc021wbw.1	chr20:30946579-31025141

Table S5: Negative data set: Cellular 5'UTR sequences of housekeeping genes (Homo sapiens) retrieved from UCSC genome browser Assembly hg19 (Total: 97)

Sr. No.	Gene name	UCSC ID[3]	Genome position
1	ENSA	uc001eve.3	chr1:150601947-150602098
2	ERH	uc001xlc.2	chr14:69864951-69865021
3	EXOC2	uc003mte.4	chr6:637819-693141
4	FH	uc001hyx.3	chr1:241683023-241683085
5	FPGS	uc004bsh.1	chr9:130565154-130569535
6	GALT	uc003zvf.4	chr9:34646586-34647205
7	GPI	uc002nvi.2	chr19:34884172-34884920
8	H1FX	uc003elx.3	chr3:129034746-129035120
9	HADHA	uc010yks.2	chr2:26461855-26467594
10	HNRNPH1	uc003mkh.4	chr5:179042547-179051670
11	IAH1	uc002qzt.3	chr2:9614670-9621470
12	KANSL12	uc001rry.2	chr12:49046995-49076035
13	LARP1	uc021ygh.2	chr5:154134889-154172232
14	LPIN1	uc002rbs.4	chr2:11864460-11881545
15	LSG1	uc003fui.3	chr3:194392892-194393206
16	LSS	uc002zik.2	chr21:47647545-47648738
17	MAEA	uc011bvd.2	chr4:1303599-1305841
18	MAK16	uc010lvu.1	chr8:33330904-33358008
19	MAVS	uc002cvv.3	chr16:3929918-3930121
20	MAX	uc031qpb.1	chr14:65541842-65569413
21	MAZ	uc002duc.1	chr16:29819966-29823306
22	MCU	uc001jtd.3	chr10:74452377-74452821
23	MED4	uc010tgf.2	chr13:48664542-48669277
24	MEPCE	uc003uuv.3	chr7:100026413-100029048
25	ATAD3A	uc001agb.2	chr1:1447910-1451423
26	AKR7A2	uc001bbw.3	chr1:19638619-19638640
27	AKIRIN1	uc001ccw.3	chr1:39456916-39457052

28	ARNT	uc001evr.2	chr1:150849044-150849244
29	C1orf43	uc001fei.2	chr1:154192884-154193273
30	APOA1BP	uc001fpk.3	chr1:156561558-156562174
31	ARV1	uc001huh.3	chr1:231114823-231114851
32	ATE1	uc001lfr.4	chr10:123502625-123688217
33	PDHX	uc001mvt.3	chr11:34937677-34938202
34	AIP	uc001olv.3	chr11:67250505-67250629
35	ARCN1	uc001ptq.3	chr11:118443102-118443262
36	PLEKHA8P1	uc001rom.2	chr12:45568149-45609789
37	APPL2	uc001tlg.1	chr12:105593276-105630008
38	APEX1	uc001vxg.3	chr14:20923290-20923804
39	ALKBH1	uc001xuc.1	chr14:78174348-78174356
40	ARIH1	uc002aut.4	chr15:72766667-72766980
41	BBS4	uc002avc.3	chr15:72978520-73016925
42	ALYREF	uc002kbu.2	chr17:79849457-79849462
43	ASPSCR1	uc002kdb.1	chr17:79936062-79941502
44	ATP5D	uc002lrn.3	chr19:1241749-1241849
45	AES	uc002lwy.1	chr19:3062199-3062371
46	CHMP2A	uc002qti.3	chr19:59065580-59066005
47	COX7A2L	uc002rsl.3	chr2:42577645-42596150
48	AFTPH	uc002scz.3	chr2:64751439-64778608
49	COA5	uc002syz.3	chr2:99224869-99224981
50	AGFG1	uc002vpd.2	chr2:228336888-228337137
51	AF055024	uc002vyh.3	chr2:239359013-239360880
52	AHCY	uc002xaj.3	chr20:32883336-32899608
53	CSTB	uc002zdr.4	chr21:45196151-45196256
54	C21orf33	uc002zed.4	chr21:45553494-45553579
55	AP1B1	uc003afh.3	chr22:29727806-29728310
56	AX747758	uc003apb.1	chr22:36633473-36635231
57	ADSL	uc003ayp.4	chr22:40742504-40742562
58	SUMF1	uc003bps.2	chr3:3823036-4508966
59	BTF3	uc003kcr.1	chr5:72794250-72794492
60	AGGF1	uc003kes.3	chr5:76326210-76326591
61	COX7C	uc003kir.3	chr5:85913784-85913872
62	ANXA6	uc003lto.2	chr5:150501816-150537443
63	AGPAT1	uc003oag.3	chr6:32139254-32145119
64	AMD1	uc003pul.1	chr6:111195987-111213380
65	ARPC1A	uc003upx.2	chr7:98923496-98930976
66	FSCN3	uc003vmc.1	chr7:127231463-127231996
67	APTX	uc003zrl.3	chr9:32985975-32990086
68	APOOL	uc004eem.3	chrX:84258898-84258918
69	ATP6AP1	uc004flh.1	chrX:153657191-153657352
70	CAPZB	uc009vpk.3	chr1:19775409-19812066
71	CC2D1A	uc010dzh.2	chr19:14023981-14029952

72	AK2	uc010ohr.2	chr1:33490118-33502512
73	APH1A	uc010pbz.2	chr1:150240126-150241609
74	AKIP1	uc010rbs.2	chr11:8932739-8932996
75	API5	uc010rfh.1	chr11:43333505-43333677
76	AMBRA1	uc010rgt.2	chr11:46564265-46564531
77	ARFGAP2	uc010rhc.2	chr11:47193217-47198676
78	ARL1	uc010svo.2	chr12:101786903-101801572
79	AHSA1	uc010tvk.1	chr14:77924373-77924532
80	KLC1	uc010tyd.1	chr14:104029299-104029338
81	ANP32A-IT1	uc010uka.2	chr15:69098985-69099440
82	AGPS	uc010zfb.1	chr2:178257769-178285006
83	CANX	uc011dgq.2	chr5:179125930-179135259
84	ARMC1	uc011leo.2	chr8:66539518-66546452
85	ALAD	uc011lxe.2	chr9:116155840-116163400
86	ARAF	uc011mlo.3	chrX:47420499-47424441
87	AKR1A1	uc021omx.1	chr1:46016455-46027466
88	BLOC1S1-RDH5	uc021qyt.1	chr12:56109818-56115573
89	C12orf65	uc021rft.1	chr12:123718028-123738221
90	ASXL1	uc021wbw.1	chr20:30946147-30946578
91	CTBP1	uc031sdc.1	chr4:1209779-1237041
92	ANAPC10	uc031shn.1	chr4:145915727-146019371
93	AAAS	uc001scr.4	chr12:53715250-53715412
94	DDX21	uc001jow.2	chr10:70716196-70719678
95	DPH1	uc031qxv.1	chr17:1943966-1945341
96	ECI1	uc002cps.3	chr16:2301568-2301602
97	FAM178A	uc001krq.4	chr10:102672326-102672867

Table S6: List of 27 small subunit ribosomal proteins used to find the interaction probabilities of 5'UTR sequences using RPISeq[4]

Sr. No.	Protein Name	Uniprot ID[5]
1.	Rps2	P15880
2.	Rps3	P23396
3.	Rps3a	P61247
4.	Rps4	P62701
5.	Rps6	P62753
6.	Rps7	P62081
7.	Rps8	P62241
8.	Rps9	P46781
9.	Rps11	P62280
10.	Rps12	P25398
11.	Rps13	P62277
12.	Rps14	P62263
13.	Rps15	P62841
14.	Rps15a	P62244

15.	Rps16	P62249
16.	Rps17	P08708
17.	Rps18	P62269
18.	Rps19	P39019
19.	Rps20	P60866-2
20.	Rps23	P62266
21.	Rps24	P62847
22.	Rps26	P62854
23.	Rps27	P42677
24.	Rps27a	P62979
25.	Rps28	P62857
26.	Rps29	P62273-2
27.	Rps30	P62861

References

1. Mokrejs, M., Masek, T., Vopalensky, V., Hlubucek, P., Delbos, P. and Pospisek, M. (2010) IRESite--a tool for the examination of viral and cellular internal ribosome entry sites. *Nucleic Acids Research*, **38**, D131-136.
   Available at http://iresite.org/IRESite_web.php

2. Benson, D.A., Cavanaugh, M., Clark, K., Karsch-Mizrachi, I., Lipman, D.J., Ostell, J. and Sayers, E.W. (2013) GenBank. *Nucleic Acids Research*, **41**, D36-D42.
   Available at http://www.ncbi.nlm.nih.gov/genbank/

3. Kent, W.J., Sugnet, C.W., Furey, T.S., Roskin, K.M., Pringle, T.H., Zahler, A.M., Haussler and David. (2002) The Human Genome Browser at UCSC. *Genome Research*, **12**, 996-1006.
   Available at http://genome.ucsc.edu/index.html

4. Muppirala, U.K., Honavar, V.G. and Dobbs, D. (2011) Predicting RNA-protein interactions using only sequence information. *BMC Bioinformatics*, **12**, 489
   Available at http://pridb.gdcb.iastate.edu/RPISeq/index.html

5. The UniProt, C. (2015) UniProt: a hub for protein information. *Nucleic Acids Research*, **43**, D204-D212.
   Available at http://www.uniprot.org/
